# Anticipation of Scheduled Feeding in BTBR Mice Reveals Independence and Interactions Between the Light- and Food-Entrainable Circadian Clocks

**DOI:** 10.3389/fnint.2022.896200

**Published:** 2022-05-30

**Authors:** Jhenkruthi Vijaya Shankara, Ralph E. Mistlberger, Michael C. Antle

**Affiliations:** ^1^Department of Psychology, University of Calgary, Calgary, AB, Canada; ^2^Hotchkiss Brain Institute, Cumming School of Medicine, University of Calgary, Calgary, AB, Canada; ^3^Department of Psychology, Simon Fraser University, Burnaby, BC, Canada; ^4^Department of Physiology and Pharmacology, Cumming School of Medicine, University of Calgary, Calgary, AB, Canada

**Keywords:** anticipation, feeding, coupling, circadian, non-photic

## Abstract

Many animal species exhibit food-anticipatory activity (FAA) when fed at a fixed time of the day. FAA exhibits properties of a daily rhythm controlled by food-entrainable circadian oscillators (FEOs). Lesion studies indicate that FEOs are separate from the light-entrainable circadian pacemaker (LEP) located in the suprachiasmatic nucleus. While anatomically distinct, food- and light-entrainable clocks do appear to interact, and the output of these clocks may be modulated by their phase relation. We report here an analysis of FAA in the BTBR T^+^ Itpr3^tf^/J (BTBR) mouse strain that provides new insights into the nature of interactions between food- and light-entrained clocks and rhythms. BTBR mice fed *ad libitum* exhibit an unusually short active phase and free-running circadian periodicity (~22.5 h). In a light-dark cycle, BTBR mice limited to a 4 h daily meal in the light period show robust FAA compared to the C57BL/6J mice. In constant darkness, BTBR mice exhibit clear and distinct free-running and food-anticipatory rhythms that interact in a phase-dependent fashion. The free-running rhythm exhibits phase advances when FAA occurs in the mid-to-late rest phase of the free run, and phase delays when FAA occurs in the late active phase. A phase-response curve (PRC) inferred from these shifts is similar to the PRC for activity-induced phase shifts in nocturnal rodents, suggesting that the effects of feeding schedules on the LEP in constant darkness are mediated by FAA. A phase-dependent effect of the free-running rhythm on FAA was evident in both its magnitude and duration; FAA counts were greatest when FAA occurred during the active phase of the free-running rhythm. The LEP inhibited FAA when FAA occurred at the end of the subjective day. These findings provide evidence for interactions between food- and light-entrainable circadian clocks and rhythms and demonstrate the utility of the BTBR mouse model in probing these interactions.

## Introduction

In mammals, the circadian pacemaker in the suprachiasmatic nuclei (SCN) orchestrates daily endogenous rhythmicity and stable entrainment to external light-dark (LD) cycles (Antle and Silver, 2005). As such, the SCN clock is known as the Light-Entrainable Pacemaker (LEP). Outputs from the LEP control a variety of endocrine, physiological, and behavioral rhythms, each of which can be influenced by various peripheral oscillators that coordinate tissue- and organ-specific rhythmicity (Hastings et al., 2019). Notable among these are rhythms that influence feeding and digestion. When food is freely available, nocturnal animals eat primarily at night, and digestion, absorption, and elimination rhythms are phased to optimally utilize ingested food and nutrients (Mistlberger and Antle, 2011).

While light is a dominant zeitgeber for the circadian system in most species, food and feeding also influence the circadian system (Stephan, 2002; Mistlberger and Antle, 2011). Nocturnal rodents who are restricted to one daily meal in the middle of the light period (normally the rest phase of the circadian rest-activity cycle), become active in advance of mealtime, a phenomenon known as food-anticipatory activity (FAA, Mistlberger, 1994; Stephan, 2002). Anticipation of a daily meal exhibits canonical properties of a rhythm generated by circadian oscillators entrainable by periodic food availability (Boulos and Terman, 1980; Mistlberger, 1994; Stephan, 2002). As FAA is robust in rodents with complete ablation of the SCN, food-entrainable oscillators (FEOs) responsible for FAA must be located elsewhere in the brain or body (Stephan et al., 1979a,b; Boulos et al., 1980; Abe and Rusak, 1992; Marchant and Mistlberger, 1997). There are multiple candidate sites, because circadian oscillators in some brain areas, and most body tissues, entrain preferentially to cycles of food availability, changing their alignment with the SCN pacemaker, which remains entrained to the LD cycle (Damiola et al., 2000; Hara et al., 2001; Stokkan et al., 2001; Wakamatsu et al., 2001).

While it is clear that the LEP and FEOs are separate and distinct, these clocks are likely coupled (Stephan, 1986a). The LEP is commonly described as both critical for entrainment to LD cycles and not affected by daily feeding schedules. However, there is ample evidence that it is affected by feeding schedules in some species, or under some conditions. In rats and mice entrained to LD cycles, there may be no detectable change in the SCN clock phase when food access is limited to the middle of the light period, as indicated by the timing of nocturnal activity unmasked in constant lighting conditions (Rosenwasser et al., 1984; Stephan, 1986a), or by the timing of clock gene rhythms in the SCN (Damiola et al., 2000; Hara et al., 2001; Stokkan et al., 2001; Wakamatsu et al., 2001). However, there are reports that the LEP phase can be advanced, particularly if mealtime occurs during the last 6 h of the light period, and when the amount of food provided is hypocaloric and weight-reducing (Mendoza et al., 2005; Pavlovski et al., 2018; Power and Mistlberger, 2020). In DD or constant dim light, free-running rhythms in various species can stably entrain to a daily mealtime, or exhibit shifts in phase (Gibbs, 1979; Kennedy et al., 1991, 1996; Mistlberger, 1991; Abe and Rusak, 1992; Castillo et al., 2004; Abe et al., 2007). Susceptibility to these effects varies by species (e.g., more common in mice than rats) and may vary within species by strain. Although this has been described as “food entrainment,” the nature of the zeitgeber is an open question. In addition to food intake, daily feeding schedules are also associated with anticipatory activity and behavioral arousal, and with changes in body temperature and various cellular and systemic metabolic signals. The LEP, *in vivo*, may be resistant to shifting in response to temperature or metabolic signals in the physiological range (Buhr et al., 2010; Crosby et al., 2019), but it can be shifted and entrained by daily schedules of forced or voluntary activity (Mrosovsky, 1996) or arousal (Webb et al., 2014). Shifting or entrainment of the LEP by feeding schedules could therefore be a response to the daily bout of FAA. If so, then shifts induced by feeding schedules should be similar to shifts induced by activity schedules, where similarity can be defined by the relationship between the timing of the stimulus (mealtime or exercise time) and the size and direction of any phase shift, which can be visualized as a phase-response curve (PRC).

Interactions between light- and food-entrainable circadian rhythms can also operate in the opposite direction, with the phase of the LEP influencing the expression of FEO-controlled FAA. The duration and amplitude of FAA may also be influenced by the phase relationship with the LEP rhythm (Stephan, 1986a) such that the FAA levels may be enhanced when feeding is in phase with the active phase of the LEP -controlled rest-activity cycle, compared to the rest phase (Petersen et al., 2014).

Recently, we have described an interesting circadian phenotype in the inbred BTBR T^+^ Itpr3^tf^/J (BTBR) mouse strain (Vijaya Shankara, 2019; Vijaya Shankara et al., in press). The BTBR strain was derived in the mid-twentieth century. Its name derives from its Black and Tan phenotype (BT, nonagouti, a^t^), and its allele for the Brachyury protein (BR, T^+^). It also carries a tufted phenotype that derives from its allele for the inositol triphosphate receptor 3 (Itpr3^tf^). The BTBR strain has been maintained in recent years as a mouse model for research on autism spectrum disorder. Specifically, BTBR mice are less social, exhibit less exploratory behavior, are more anxious, and exhibit altered ultrasonic vocalizations (Mcfarlane et al., 2008; Meyza et al., 2015; Meyza and Blanchard, 2017). Of interest to circadian studies, these mice have very short free-running periods (τ = ~22.5 h) in DD and short active phases (α). This may make the BTBR mouse a useful model for studying interactions between LEP-mediated free-running rhythms and FEO-mediated anticipation of daily feeding schedules. Although the short τ should decrease the probability that the free-running rhythm will entrain to a 24 h feeding schedule in DD, it will increase the opportunity to detect repeated modulations of free-running and FAA rhythm parameters, within individual mice, as a function of the phase relationship between mealtime and the free-running rhythm.

## Materials and Methods

### Animals

A total of six male BTBR T^+^ Itpr3^tf^/J (BTBR) and six male C57BL/6J (B6) were used in this study. One B6 mouse experienced persistent equipment failure during the LD portion of the study and was therefore included only in the DD part of the study. B6 mice were used as a comparison strain in this study as they are widely used in circadian studies, including those involving food restriction (Marchant and Mistlberger, 1997; Power and Mistlberger, 2020; Mei et al., 2021). All mice were obtained from Dr. J. Rho at the Alberta Children's Hospital Research Institute, University of Calgary, with founding animals obtained from Jackson Labs (#:002282). Male BTBR mice were used as they tend to have shorter periods than female BTBR mice (Vijaya Shankara, 2019; Vijaya Shankara et al., in press). The mice were at least 3 weeks old and 20 g in weight when received and between 3 and 8 months of age during experiments. Animals had *ad libitum* access to food (Purina Lab Diet 5001) at all times except during the scheduled feeding protocol. The mice were individually housed in clear polycarbonate cages (Nalgene Type L) equipped with a 24.2 cm diameter stainless steel running wheel. Running wheels were equipped with magnetic switches and were connected to a computer running the Clocklab Data Collection software (Actimetrics, Wilmette, IL, United States). Actograms were plotted as the “normalized” type.

### Procedures

#### Food Restriction Protocol

The animals were first allowed to stably entrain to a 12:12 LD cycle for 3 weeks. The light intensity was ~200 lux measured at cage level and was provided by overhead fluorescent lights. After an overnight fast, the restricted feeding (RF) schedule was initiated with food availability starting at Zeitgeber Time (ZT) 4 (i.e., 4 h after lights-on, where ZT12 is lights-off, by convention). Food was available for 16 h on day 1 and then reduced to 12 h on day 2, 8 h on day 3, 6 h on day 4, and finally 4 h per day (ZT4-ZT8) for the next 2 weeks. Food was then made available *ad libitum* for the next 3 weeks in LD and another 3 weeks in DD, after which food availability was restricted to 4 h per day, beginning at 12:00 h local time, for 7 more weeks in DD. Food was manually delivered and removed by the experimenter with the aid of night-vision goggles.

#### Data Analysis in LD

The activity levels were calculated by averaging activity over 7 days using the Clocklab analysis software (Actimetrics, Wilmette, IL, United States). For analysis of baseline activity, the last 7 days before restricted feeding (RF) were used, while analysis of activity during RF used 7 days of data starting on the 5th day of the RF protocol (the first day with a 4 h food window, and by which time all animals were anticipating). The average activity profiles were generated in Clocklab over these days. The following were quantified: daily activity, activity in the anticipation window (ZT0-4), and the anticipation ratio (ZT0-4 activity/total daily activity). FAA onset was defined as the first of two or more consecutive 10 min bins between ZT0-4 with at least 10 activity counts/min. FAA duration was then calculated as the time from FAA onset to mealtime. Independent samples *t*-tests were used to examine the difference between BTBR and B6 mice. Paired *t*-tests were used to examine the differences between total daily activity during baseline and RF within each strain.

The nocturnal activity was quantified by summing counts in the early-night (ZT12-16), mid-night (ZT16-20), and late-night (ZT20-24), for the 7 baseline days and RF days 5–12. Nocturnal activity onsets and offsets were also identified by Clocklab over these day ranges as well as for days 4–11 of *ad libitum* recovery feeding. The duration of nocturnal activity (α) was calculated as the time between Clocklab-identified activity onset and offset. A two-way repeated measures ANOVA was used to examine nocturnal activity levels between baseline and RF over the early-, mid-, and late-night time blocks. Differences in α onset, offset, and duration were analyzed using two-way factorial ANOVAs comparing the variables between the strains and across the baseline and RF periods and post-RF recovery periods. All means are reported ± StDev in the text and ± SEM in the figures.

#### Data Analysis in DD

While B6 mice were subjected to the RF schedule in DD, the analysis focused primarily on the BTBR mice.

Baseline free-running period, average waveforms, and alpha duration were calculated in 4 BTBR mice and 5 B6 mice over 10 days starting 2 weeks into the DD period. Equipment failure prevented analysis from 2 BTBR mice and 1 B6 mouse during this period. The period was assessed in Clocklab using the chi-squared periodogram. Average waveforms were created using the activity profile routine in Clocklab with the period set to that calculated from the chi-squared periodogram. The raw data from these individual average waveforms were loaded into a spreadsheet and aligned such that their activity onsets (1st bin with >20% of max activity observed in any bin) occurred on the same row. The values were then averaged across all animals to generate the Average Waveform for each group. The activity duration (alpha) for individual animals was determined from the activity profile data in the spreadsheet. Specifically, the 10-min bin with the maximum activity was identified, and alpha was defined as the period that activity remained above 20% of this max activity value, allowing for gaps of up to 1 h. The offset was the last bin of >20% max activity that was preceded by at least 4/5 other such bins. Alpha was the time between onsets and offsets defined in this fashion.

##### Measuring Phase Shifts of Free-Running Rhythms During Restricted Feeding

To determine if FAA might phase shift or entrain the free-running LEP in DD, actograms were plotted modulo-τ of the free-running rhythm (22.5–22.8 h, quantified during baseline DD using the Lomb-Scargle periodogram routine in Clocklab). The phase shifts were quantified by fitting regression lines to activity onsets for blocks of days before and after the day exhibiting the phase shift. The activity onsets were identified using the Clocklab algorithm, with the on/off times initially set to 1 h and 7 h, respectively. The timing of FAA and food delivery relative to circadian time (CT) of the free-running rhythm (where CT12 is the onset of the main active phase, or “subjective night,” of the LEP-controlled free-running rest-activity cycle) were determined. The relationships between phase shifts and the CT of FAA, the amount of FAA, and the CT of mealtime were explored using Pearson correlations. The free-running period before and after the phase shift was determined by the slope of the regression lines. Differences in phase shifts induced by FAA during the mid-late subjective day versus the late subjective night were examined with a Student's *t*-test.

##### Measuring FAA Magnitude at Different Phases of the Free-Running Rhythm in DD

To determine if the amount of FAA was influenced by the phase of the LEP in DD, activity in the 4 h preceding the daily meal was summed and averaged within four different time blocks, or phase zones of the free-running rhythm. The four-phase zones were defined based on mealtime relative to CT of the free run (see Table 1). Specifically, we examined properties of FAA when activity onset of the LEP fell in the following 4 windows: during the 4 h FAA window, during a 6-h window starting with food delivery, during the 6-h window following the feeding window, and during the 8-h window prior to the FAA window. When collected in this manner, some windows did receive more samples than others, but as these were averaged together, each animal still only contributed one data point to each window. Due to the short free-running periods, if an animal had CT12 occurring during both the pre-FAA and post-feeding windows, the FAA on that day was allocated to the post-feeding window. In addition to the amount of FAA, a measure of FAA consolidation was derived by counting the number of pauses in activity (10 min or more) after FAA onset. A one-way repeated measures analysis of variance was used to test for differences in FAA amount and consolidation across the four-phase zones of the free-running rhythm. The Holm-Sidak test was used for pairwise *post-hoc* comparisons.

**Table 1:** Categories for phase relationships between the FEOs and LEP during restricted feeding under constant darkness.

**Clock time of CT12**	**CT of FAA onset**	**CT of food onset**	**LEP-FEO relationship**
00:00-8:00	CT12-20	CT16-24	LEP onset in advance of FAA
8:00-12:00	CT8-12	CT12-16	LEP onset within FAA window
12:00-18:00	CT0-6	CT6-12	LEP onset within feeding window
18:00-24:00	CT20-2	CT0-6	LEP onset follows feeding window

*Food was available between 12:00 and 16:00 local clock time. The colors correspond to the colors used in Figure 5*.

##### Suppression of FAA by the LEP

Visual inspection of the actograms suggested that FAA onset was abruptly delayed on the day that CT12 of the free-running rhythm coincided with meal onset. To quantify this apparent reduction in FAA duration, Clocklab-identified activity onsets were identified for the LEP when they preceded the feeding window. A regression line was fit to these onsets and was extrapolated back to determine the first day that CT12 of the LEP fell within the FAA window and before food presentation. The onset times of FAA on the 4 days preceding this were identified by Clocklab as described above and were averaged to obtain an average FAA onset time. These were compared to the actual activity onset on day 5 (i.e., the day when CT12 of the LEP first appeared within the FAA window and before food delivery). The amount of activity from each identified onset until food delivery was quantified. For the baseline FAA days (days 1–4) this activity was averaged. One-way ANOVAs were used to determine if (1) FAA occurred later on the day that CT12 of the LEP first appeared in the FAA window, and (2) if the activity levels during FAA were lower on this day. All means are reported ± StDev in the text and ± SEM in the figures.

## Results

### In LD BTBR and B6, Mice Exhibit Robust FAA and Altered Nocturnal Activity

**Figure 1:**
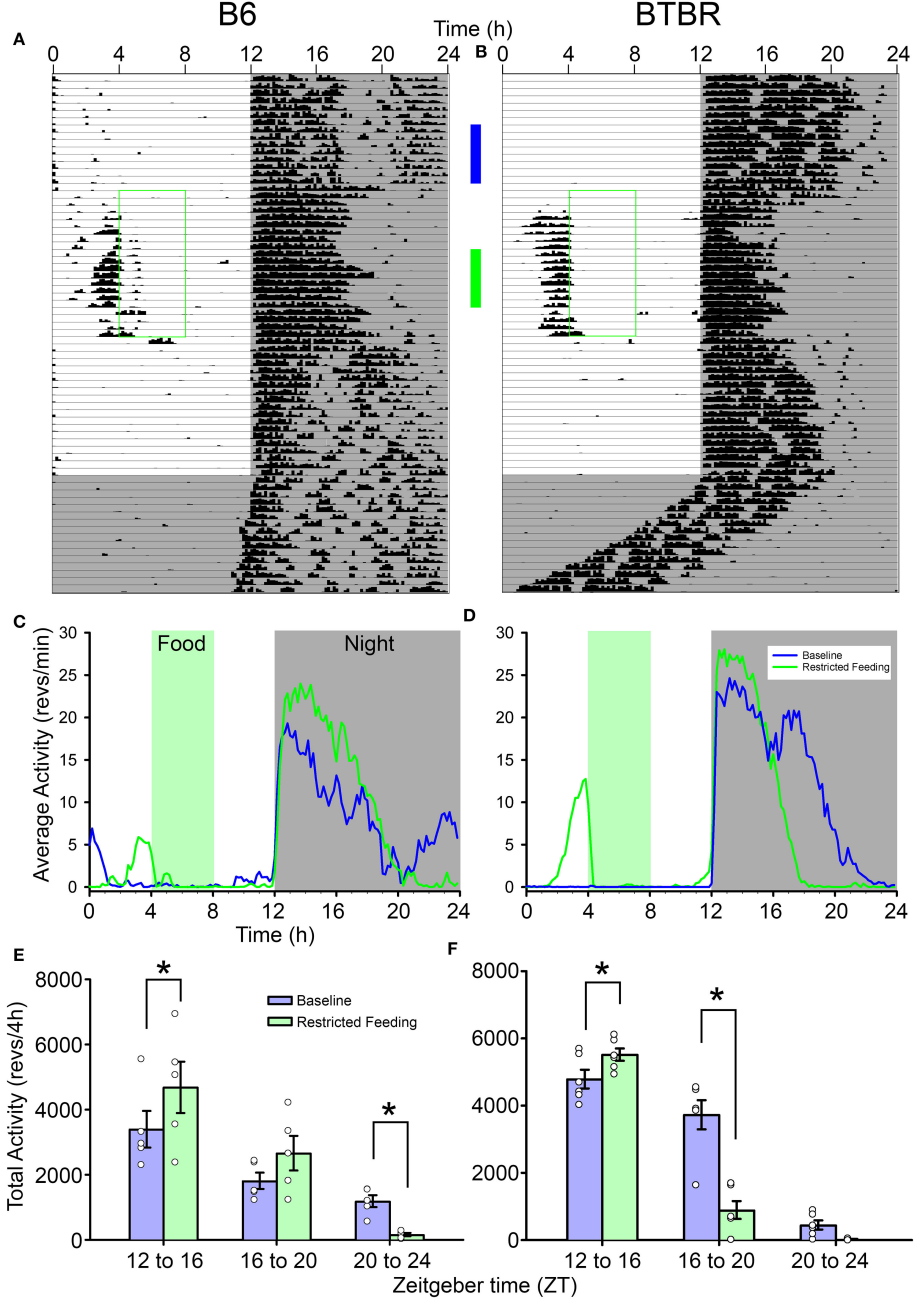
Representative actograms from **(A)** a B6 mouse and **(B)** a BTBR mouse. The green box on each indicates the time of restricted food access. The vertical blue and green bars between the actograms indicated the approximate days used for the analyses presented in **(C–F)**. Average waveforms from 7 days of activity during baseline (blue) and restricted feeding (green) are presented for B6 mice **(C)** and BTBR mice **(D)**. The green rectangle denotes the time of food access during the restricted feeding portion of the study. The average total activity in the early-night (ZT12-16) mid-night (ZT16-20), and late-night (ZT20-24) are presented for both baseline (blue) and restricted feeding (green) for both B6 **(E)** and BTBR **(F)** mice. Activity levels in the early-night are significantly increased when on restricted feeding, while activity levels are significantly decreased in the mid-night for BTBR mice and the late-night for B6 mice. *p < 0.05.

Both strains showed robust FAA to RF (Figures 1A,B). While BTBR mice tended to show slightly longer (1.74 ± 0.5 h) and more intense (1,034 ± 500 counts) FAA than did B6 mice (1.33 ± 0.8 h; 493 ± 700 counts), neither of these differences were significant [duration: *t*_(9)_ = 1.019, *p* = 0.33, activity levels: *t*_(9)_ = 1.49, *p* = 0.17]. The anticipation ratios for BTBR mice (0.13 ± 0.06) also tended to be larger than for B6 mice (0.05 ± 0.06) but this difference was not significant [*t*_(9)_ = 2.047, *p* = 0.07].

The nocturnal activity of both strains was altered by RF. While nocturnal activity onset did not change from baseline [*F*_(2, 18)_ = 1.181, *p* = 0.33], the offsets were significantly earlier during RF [*F*_(2, 18)_ = 25.678, *p* < 0.001], resulting in a significantly shorter nocturnal α [*F*_(2, 18)_ = 21.328, *p* < 0.001]. Consistent with previous observations, α was significantly shorter in the BTBR mice than in B6 mice [main effect of strain, *F*_(2, 18)_ = 9.861, *p* = 0.012]. There was no significant interaction between strain and phase of the study [*F*_(2, 18)_ = 2.275, *p* = 0.132], indicating that the significantly shorter α in BTBR mice was evident whether the food was available *ad libitum* or time-restricted. Offsets and α returned to baseline values following the end of RF.

The reorganization of nocturnal activity also led to alterations in nocturnal activity levels within each strain (Figures 1C–F). There were significant interactions between treatment (baseline vs. RF) and nocturnal phases (early-, mid-, and late-night) for both BTBR [*F*_(2, 10)_ = 25.155, *p* < 0.001] and B6 mice [*F*_(2, 8)_ = 6.344, *p* = 0.022]. In both strains, activity levels in the early-night were significantly increased during RF relative to baseline. The activity levels of BTBR mice were significantly suppressed in the middle of the night during RF relative to baseline, while there was no difference in the late-night when BTBR mice typically have little activity during baseline. For B6 mice, there was no difference in activity levels in the middle of the night during RF, but activity in the late-night was significantly suppressed relative to baseline.

### Phase-Dependent Phase Shifts of the Free-Running Rhythm During Restricted Feeding in DD

**Figure 2:**
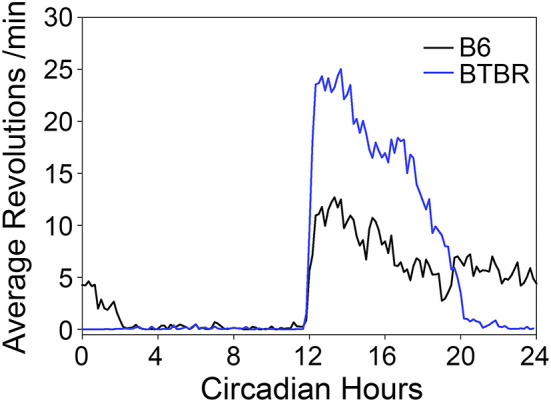
Average waveforms of wheel-running activity rhythms in DD.

During the baseline period in DD prior to RF, BTBR mice exhibited shorter free-running periods [22.9 ± 0.2 h) than did B6 mice (23.9 ± 0.09 h, *t*_(7)_ = 9.53, *p* < 0.0001]. Average waveforms (Figure 2) revealed a similar pattern to that observed in LD, with BTBR mice exhibiting greater amounts of activity in the early subjective night. BTBR mice also had significantly shorter α (6.84 ± 1.35 h) relative to the B6 mice [11.97 ± 2.26 h; *t*_(7)_ = 3.98, *p* = 0.005].

**Figure 3:**
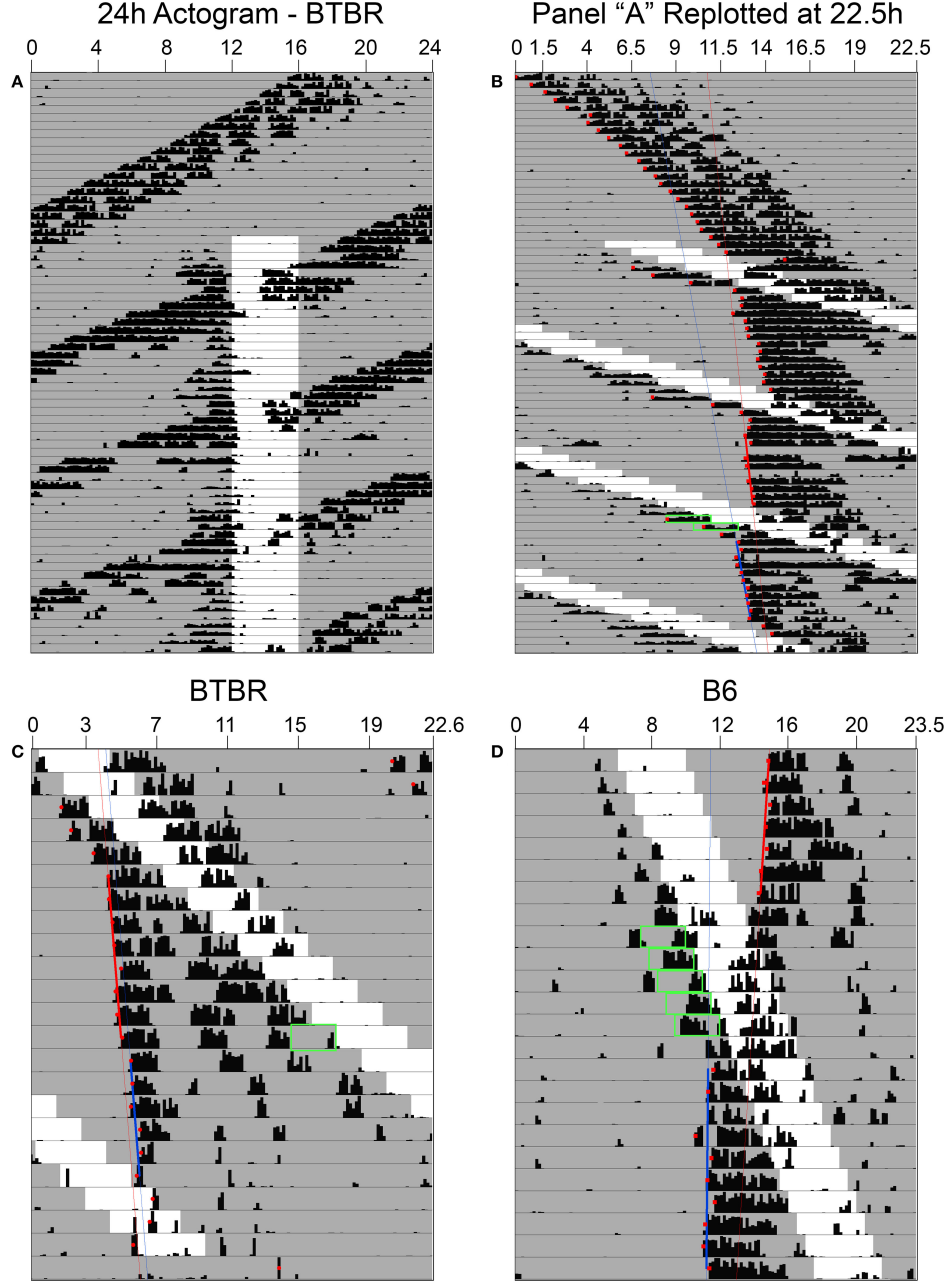
In constant darkness, activity controlled by the light-entrainable pacemaker (LEP) in BTBR mice free-runs through the restricted feeding schedule **(A)**. When actograms are re-plotted relative to the free-running period of the LEP **(B)**, phase shifts of these rhythms are revealed when food-anticipatory activity (FAA) occurs in the mid-late subjective day for the LEP. **(C)** When FAA occurs in the late subjective night, phase delays are observed. **(D)** Phase shifts to the LEP are also apparent in B6 mice when the free-running LEP intersects with the schedule feeding time, although given their slower free-running period relative to BTBR mice, they exhibit FAA in the mid-late subjective day over numerous consecutive days. Green boxes highlight FAA bouts that occurred on days associated with a shift at phases consistent with previously published PRCs for non-photic phase shifts.

**Figure 4:**
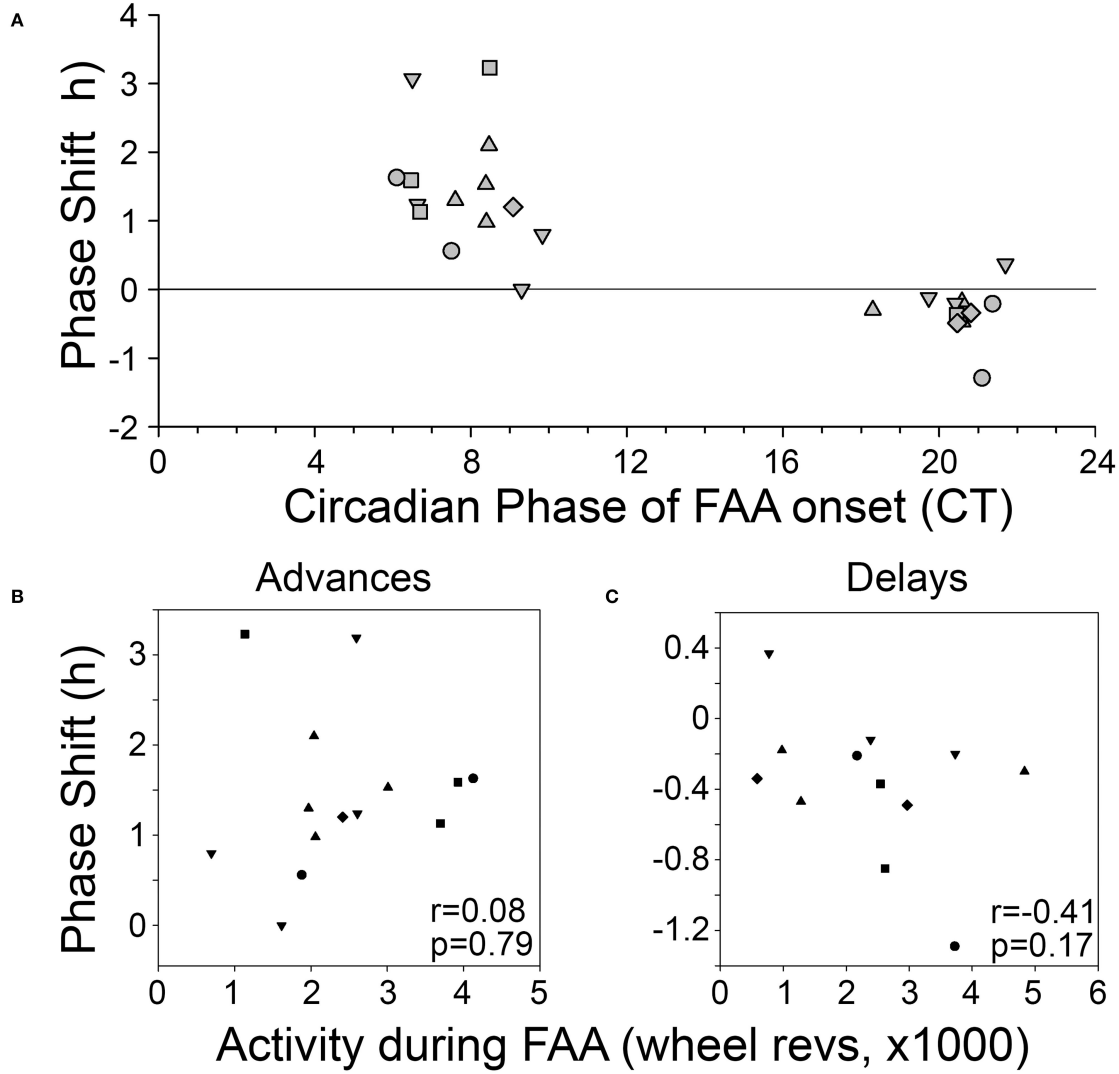
**(A)** Phase-response curve for phase shifts induced by FAA. Correlations between the amount of activity during FAA and the size of the resulting phase advance **(B)** or phase delay **(C)**. Each animal is represented by a different symbol.

Phase shifts of the free-running rest-activity cycle were apparent in all BTBR animals, often multiple times throughout the complete record (Figures 3A,B). Across all 6 mice, there were 14 instances where FAA began during the mid-late subjective day and when activity records could be clearly assessed to quantify phase shifts. The average phase shift was 1.45 ± 0.88 h (range = 0 to 3.23 h). The average FAA onset phase was CT7.8 ± 1.2 (range = CT6.47–CT9.83). This corresponds to an average meal onset of CT10.55 ± 0.68. The average amount of FAA in this phase range was 2412.2 ± 1,010 wheel revolutions. There was no significant correlation between the size of the phase shift and (1) FAA amount (*r* = 0.08, *p* = 0.79, Figure 4B), (2) CT of FAA within the CT6–12 zone (*r* = −0.31, *p* = 0.29), or (3) mealtime within the CT6–12 zone (*r* = −0.24, *p* = 0.40).

On average, the free-running period before the shift (22.72 ± 0.22 h) did not differ significantly from the free-running period after the shift [22.63 ± 0.16 h, *t*_(24)_ = 1.14, *p* = 0.27].

Free-running τ in DD prior to the onset of RF in the B6 mice averaged 23.8 ± 0.16 h, ~1 h longer than in the BTBR mice (22.88 ± 0.29 h). Consequently, mealtimes relative to CT changed much more gradually from day to day. One B6 mouse showed a large phase advance of 3.38 h when mealtime began late in the subjective day (Figure 3D), but this CT zone was not sampled sufficiently during the 7 weeks of restricted feeding to permit group analyses.

Across the 6 BTBR mice, 12 phase shifts, averaging −0.37 ± 0.39 h (range = +0.37 to −1.29 h), were evident when FAA began at CT20.5 ± 0.9 (Figure 3C). The average time of meal onset for these days was CT23.2 ± 1.1 h. The average amount of FAA at this CT was 2382.3 ± 1,318 wheel revolutions. There was no significant correlation between the size of the delay shift and (1) FAA amount (*r* = − 0.41, *p* = 0.17, Figure 4C), (2) CT of FAA onset (*r* = 0.04, *p* = 0.91), or (3) CT of meal onset (*r* = −0.17, *p* = 0.59). There was a significant difference between the phase shifts elicited when FAA began during the mid-late subjective day (CT6–10) and when it began during the mid-late subjective night [CT18–22, *t*_(24)_ = 6.6, *p* < 0.0001, Figure 4A]. The free-running period before delays shifts (22.58 ± 0.21 h) did not differ significantly from the free-running period after delay shifts [22.63 ± 0.25 h, *t*_(22)_ = 0.67, *p* = 0.51].

### Phase-Dependent Modulation of FAA Magnitude and Duration in DD

**Figure 5:**
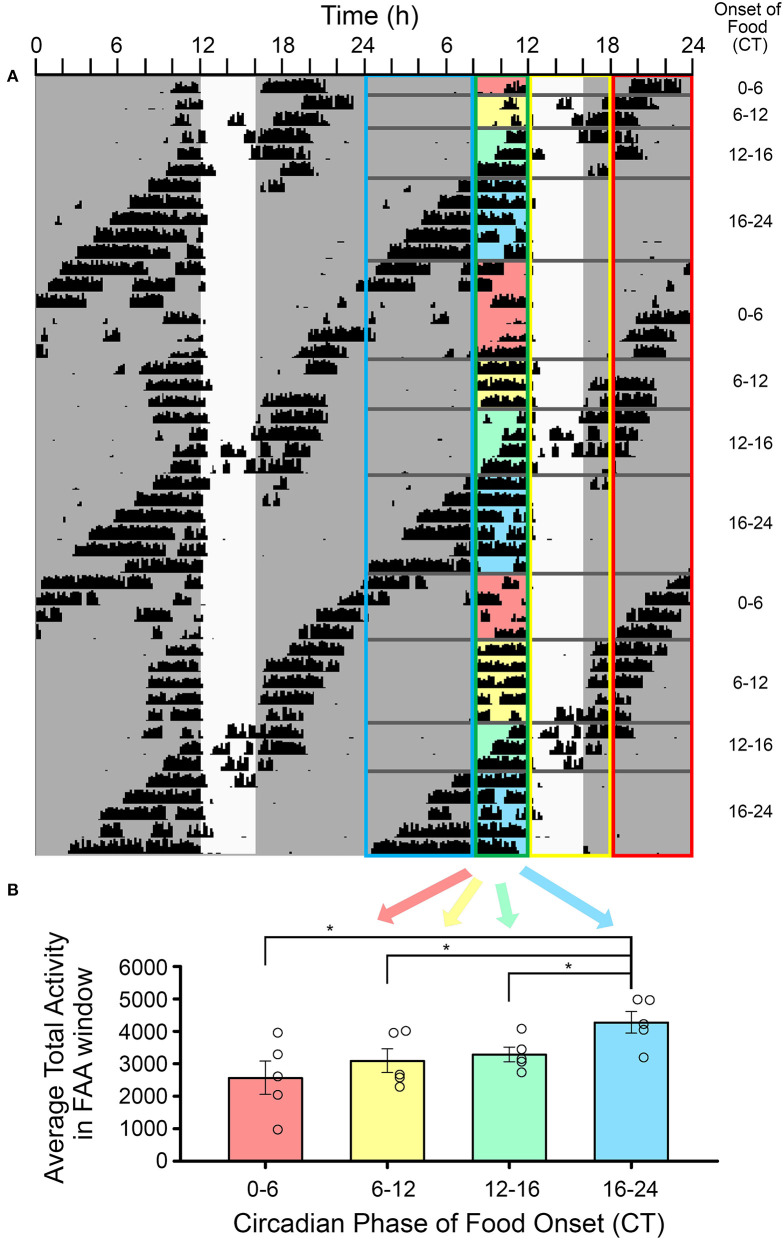
The amplitude of food-anticipatory activity (FAA) was modulated by its phase relationship with the light-entrainable rhythm. **(A)** A double-plotted actogram of a representative animal during restricted feeding in constant darkness. The white vertical rectangle denotes the time of feeding. The phase relation of activity onset (CT12) for the light-entrainable pacemaker (LEP) was assessed each day. Days were sorted into one of four categories: (1) CT12 preceded FAA, (2) CT12 occurred during the FAA window, (3) CT12 occurred during the feeding window, (4) and CT12 followed the feeding window (see Table 1). The FAA activity was summed over the 4 h preceding feeding time each day and then averaged over all days with the same phase relationship to the LEP for each animal (*n* = 5). **(B)** FAA amplitude was significantly higher when food availability started between CT16-24 (i.e., FAA started between CT12-20) than for all other phase relationships. *p < 0.05.

The average amount of FAA was significantly higher when it occurred in the early-to-mid subjective night relative to other phases of the free-running rhythm [one-way RM-ANOVA, *F*_(3, 12)_ = 6.053, *p* = 0.009, Figure 5]. FAA amount was very stable when it occurred during the late subjective day of the LEP. When CT12 for the LEP occurred during the FAA window, activity levels appeared higher, but overall anticipation duration was shorter on those days when the activity onset for the LEP was closer to the time of feeding. On days when CT12 for the LEP fell in the post-feeding period, such that the LEP and FEOs were nearly in antiphase, the timing and duration of FAA appeared more fragmented and variable. FAA that started between CT20-2 was less robust than FAA at other phases. Specifically, FAA at these phases was more fragmented, having significantly more bouts of activity during the FAA window [1.7 ± 0.24 bouts, *F*_(3, 12)_ = 3.874, *p* = 0.038] than when FAA occurred at the end of the subjective day (FAA onset between CT8-12, 1.3 ± 0.05 bouts). Additionally, FAA that started between CT20-2 also had significantly more 10 min bins with no activity [8.4 ± 4.1 bins with no activity, *F*_(3, 12)_ = 7.568, *p* = 0.004] than did FAA that fell in the early-mid subjective night (FAA onset between CT12-20, 3.0 ± 1.5 bins with no activity).

### The LEP Suppresses FAA When FAA Falls Immediately Prior to CT12

**Figure 6:**
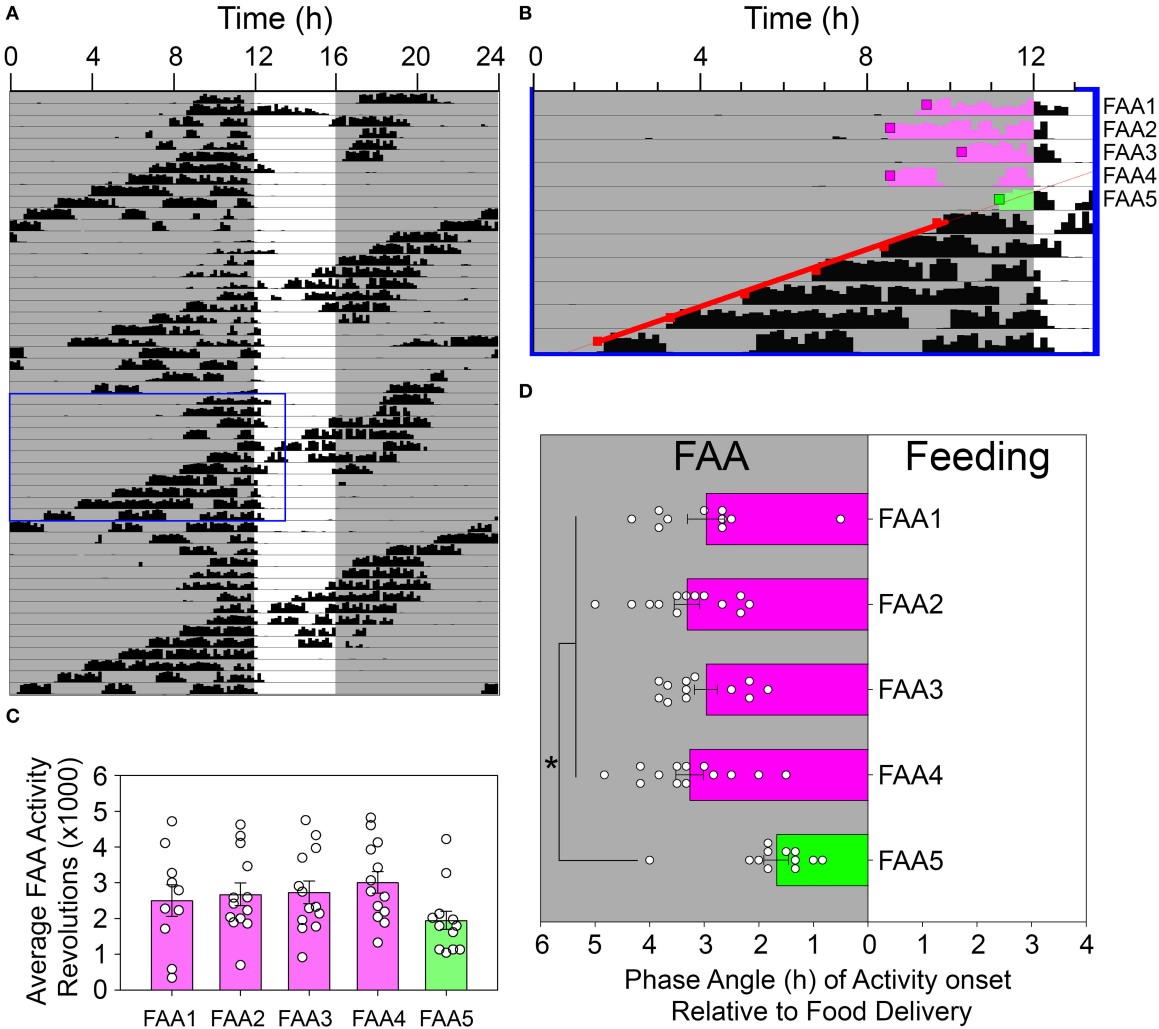
Food-anticipatory activity was suppressed on days when the rhythm controlled by the light-entrainable pacemaker (LEP) appeared in the FAA window. **(A)** An actogram depicting a representative animal during restricted feeding in constant darkness. The blue box denotes the data enlarged for presentation in **(B)**. Activity onsets were identified by Clocklab on these days. A regression line was fit to the onsets for the LEP (red squares) and extrapolated back to identify the first day that CT12 for the LEP occurred within the FAA window (green shaded activity). The onsets were determined for this day (green box) and the preceding 4 days (pink boxes). The amount of activity until feeding time **(C)** and the phase angle of activity onset relative to feeding time **(D)** were quantified and analyzed. *p < 0.05.

Visual inspection of the actograms revealed an unexpected acute reduction in FAA duration on days when the onset (i.e., CT12) of the free-running rhythm reached the meal onset (e.g., Figures 6A,B). FAA duration on this day averaged 1.68 ± 0.82 h, compared to 3.21 ± 0.66 h on the four previous days. ANOVA indicated a significant difference across these 5 consecutive days [*F*_(4, 45)_ = 10.622, *p* < 0.001, Figure 6D], with FAA duration on day 5 significantly shorter than each of the previous 4 days (Holm-Sidak *post-hoc* tests, *p* < 0.001 vs. FAA2,3,4, and *p* = 0.002 vs. FAA1). No other pairwise differences were detected. The average amount of FAA on this day was also reduced compared to the average across the 4 previous days [2,717 ± 858 revolutions vs. 1,950 ± 911 revolutions, paired *t*-test, *t*_(12)_ = 2.55, *p* < 0.025], although not when compared to each of the 4 days individually [no significant main effect of day; *F*_(4, 45)_ = 2.375, *p* = 0.068, Figure 6C].

## Discussion

The light-entrainable circadian rest-activity cycle in BTBR mice exhibits an unusually short circadian periodicity in DD and a short active phase (Vijaya Shankara et al., in press). We reasoned that these properties would make the BTBR mouse a useful model for probing interactions between light- and food-entrainable clocks and rhythms. The short τ reduces the probability that the free-running rhythm will entrain to feeding time in DD. This allows different phase relationships between food- and light-entrainable clocks and rhythms to be assessed repeatedly within a few weeks in DD in the same mice. Systematic changes in phase at particular phase relations can then be used to infer phase-response curves. The short α reduces the overlap between the behavioral outputs of these clocks, which facilitates the measurement of phase markers (e.g., α and FAA onset).

Consistent with this reasoning, our analyses of food-restricted BTBR mice in LD and DD revealed independence and interactions between light- and food-entrainable clocks and rhythms. In LD, BTBR mice showed robust FAA to a mid-day meal, with a trend toward more robust FAA compared to B6 mice. In DD, distinct free-running and 24 h FAA rhythms were evident, highlighting the independent nature of these rhythms. Notably, the free-running rhythm exhibited phase advance shifts when FAA began in the mid-late subjective day and smaller phase delay shifts when FAA occurred in the late subjective night. Furthermore, the expression of FAA varied with the phase of the free-running rhythm. The amount of FAA was significantly greater when FAA occurred during the early- to mid-active phase of the free-running rhythm, compared to other phases, suggesting that outputs of the LEP and FEOs were additive at these phases. A negative interaction was also noted, with FAA duration acutely shortened when mealtime occurred immediately following CT12 of the LEP rhythm.

### The LEP Is Reset by FAA/Feeding During the Late Subjective Day

The resetting of the LEP's phase as it intersects with the RF schedule demonstrates an influence of the FEO and/or its related inputs/outputs on the LEP. The nature of the zeitgeber, in this case, is not clear and could emerge from a combination of factors, including the direct coupling of the oscillators, effects of FAA, food, or physiological effects of the food. The similarity of the PRC here to that reported in hamsters to exercise (Mrosovsky, 1996) suggests that intense activity that accompanies anticipation could be the main cue. While the correlations between activity and the resulting shift were not significant here, we have previously demonstrated that arousal/wakefulness is a sufficient cue to induce non-photic shifts in hamsters (Antle and Mistlberger, 2000; Yamakawa et al., 2016). It is possible that the animals were significantly more alert during FAA, and that this, rather than the activity *per se*, was the critical cue. Similar non-photic PRCs are apparent in mice when inferred from entrainment (Dallmann et al., 2007), or when activity is triggered by morphine (Marchant and Mistlberger, 1995). Since FAA may manifest in a variety of behaviors, the mice here may have been highly aroused during FAA, despite the lack of wheel running. Other measures of FAA, such as bar pressing, food bin activity, or general locomotor activity (Mistlberger et al., 2009; Petersen et al., 2014), might help disentangle the contribution of arousal from intense physical activity that can only be accomplished with a running wheel. It is possible that the phase shifts were caused by ingestion of food or some downstream physiological response to food consumption. However, the timing of feeding relative to the phase of the LEP does not match known non-photic PRCs as well as does the timing of FAA. Furthermore, discrete phase shifts of the LEP have been reported to refeeding food pulses in the hamster (Mistlberger et al., 1997), and these were shown to be linked to the associated wheel-running activity rather than food ingestion. Phase shifts to these refeeding pulses exhibited phase advances in the mid-day, similar to what was observed in the present study. Recently, similar phase shifts to scheduled feeding occurring during the mid-late subjective day were reported in females, but not males, C57BL/6J mice (Mei et al., 2021). In the present study, all mice were males, including the B6 mice. While only one phase shift was noted in the B6 mice in the current study, this single mouse was also the only B6 mouse to experience FAA in the mid-late subjective day.

The magnitude of the phase shifts to FAA here is greater than has been reported in mice to other non-photic treatments. While phase shifts were calculated relative to a single day, FAA over several consecutive days might lead to an additive phase shift. This was particularly evident in the B6 mouse that also demonstrated this phenomenon. Alternatively, BTBR mice have higher amplitude locomotor rhythms, which could suggest differences in the underlying LEP. The amplitude of a pacemaker may contribute to the magnitude of observed phase shifts. Mathematically, processes with high amplitude rhythms should have smaller phase shifts to comparable perturbations, although the opposite has been observed in SCN responses to light (Ramkisoensing et al., 2014).

Entrainment of the LEP to restricted feeding has been noted previously in rats (Stephan, 1986b), but only in cases when the free-running period of the LEP was within 5 min of the period of the RF schedule. In animals with a larger period difference, while there was no entrainment to the RF schedule, relative coordination between the rhythms (i.e., modulation of the LEP period) was noted (Stephan, 1986b). This entrainment and relative coordination may have emerged from non-photic influences of the FAA through the well-documented activity/arousal influences on the LEP (Webb et al., 2014). If this is the case, certain phase relationships should be predictable. When the LEP period is slightly shorter than the period of the RF schedule, daily delays would be needed for the LEP to entrain to scheduled RF, and the FAA should occur in the late subjective night in these cases. Conversely, when the LEP period is slightly longer than the period of the RF schedule, daily advances would be needed for the LEP to entrain to scheduled RF, and the FAA should occur in the late subjective day in these cases.

### FAA Is Modulated by the Circadian Phase of the Free-Running Rhythm

The amount and duration of FAA varied with CT of the free-running rhythm. FAA counts were highest during the first ~8 circadian hours (CT12-20) of the subjective night when the LEP promotes activity and arousal. This suggests that throughout this range of phases, FAA reflects the additivity of outputs from the LEP and FEOs.

FAA parameters at other phases of the free-running rhythm provide further evidence for interaction between the food- and light-entrainable clock outputs. FAA initiated toward the end of the subjective night (CT20-CT2) appeared more variable from day to day in onset time and amount (FAA shaded red in Figure 5). BTBR mice have a short α in both LD and DD, and by ZT/CT20 they are generally inactive (Figure 1 and Vijaya Shankara et al., in press). Within this range of phases, the LEP and FEOs are in conflict, with the LEP signaling rest, and the FEOs signaling activity. As FAA onset moved toward the middle of the subjective day on subsequent days, FAA counts and duration became less variable (FAA shaded yellow in Figure 5, corresponding to FAA starting between CT2-8). This could be due to a reduction in sleep propensity, after the initial bout of sleep that marks the beginning of the subjective day. Similar interactions have been observed with FAA in response to scheduled feeding at different phases of an LD cycle (Petersen et al., 2014). In an LD cycle, FAA, as measured by motion sensors, was significantly higher when it occurred during the subjective night (i.e., when meals were initiated at ZT15, 19 and 23). The present results are consistent with these previous findings and extend them by demonstrating that the lower FAA during the day is not due to masking influences of light.

An abrupt reduction in FAA duration was noted when FAA was initiated late in the subjective day, on the days when mealtime crossed over CT12 of the free-running rhythm (Figure 6, and FAA shaded green in Figure 5). In this case, there appears to be a strong inhibition of FAA in the hours preceding CT12. This would be consistent with the suggestion that the LEP plays an active role in both promoting wake during the active phase and promoting rest during the inactive phase (Mistlberger, 2005). It should be noted that there does not appear to be any inhibition of FAA near feeding time on the preceding day when CT12 for the LEP would be within the feeding window. The exact location of CT 12 on these days is not apparent due to masking from the meal, but the lack of suppression raises the possibility that the observed suppression/interference between the LEP and FEO may be phase-dependent and is only revealed when they become sufficiently close in phase. Alternatively, the abrupt reduction in FAA duration late in the subjective day could represent an acute phase delay shift of FEOs induced by coupling inputs from the LEP, rather than suppression (masking) of FAA downstream from FEOs. This idea is consistent with other evidence that the LEP and FEOs driving FAA are mutually, albeit weakly, coupled (Stephan, 1986a; Mistlberger et al., 2001). An added complication here is that the suppression of FAA was evident on the same days that phase advances of the free-running rhythm occurred, suggesting that the observed inhibition may result from an interaction of several phenomena.

### Limitations

The similarity of FAA in BTBR and B6 mice entrained to LD indicates the normal functioning of FEOs in the BTBR strain. In DD, the B6 mice free-ran with a period length of ~23.5 h, ~1 h longer than the BTBR mice. Consequently, mealtimes relative to the phase of the free-running rhythm changed gradually, and only a limited range of phase relations between FAA and the free-running phase could be assessed in 7 weeks of restricted feeding. While only one clear phase advance shift was observed in the B6 mice, this mouse was also the only one in which FAA occurred in the mid-late subjective day. This suggests that phase-dependent phase-shifting effects of FAA on free-running rhythms are not unique to the BTBR mouse strain, but this will need to be confirmed with a larger sample of B6 mice maintained on RF for a longer duration.

### Conclusions

The results presented here provide novel evidence for interactions between food- and light-entrainable clocks and rhythms. Phase-shifting of LEP-driven free-running rhythms by daily feeding schedules is consistent with evidence that feeding schedules can entrain free-running rhythms in some species (Stephan, 1986a,c; Kennedy et al., 1991; Castillo et al., 2004; Abe et al., 2007). A PRC based on the timing of FAA relative to the free-running rhythm phase suggests that the entraining stimulus is behavioral output from FEOs, which may activate known non-photic input pathways to the SCN (Mrosovsky, 1996; Hughes and Piggins, 2012; Webb et al., 2014; Yamakawa et al., 2016; Jha et al., 2021). Apparent modulation of both LEP phase and FAA timing at certain phase relations is consistent with prior proposals that LEPs and FEOs are mutually coupled (Stephan, 1986a,b,c). Coupling strength has been described as asymmetrical, with the LEP exerting the stronger influence in rats (Stephan, 1986a), but the evidence here indicates that FEO effects on the LEP phase are considerable in mice, and may be mediated in part by the activity/arousal associated with FAA. A critical role for wheel-running activity can be evaluated in future studies by using other measures of FAA that do not involve intense locomotor activity. Finally, while the BTBR strain facilitates these investigations due to its extremely short free-running period, studies using other mouse strains and species will be needed to confirm that the phase-dependent interactions observed in BTBR mice are not unique to this strain.

## Data Availability Statement

The datasets presented in this study can be found in online repositories. The name of the repository and accession number can be found at: PRISM Dataverse, https://dataverse.scholarsportal.info/dataverse/calgary, doi: 10.5683/SP3/GRCYIO.

## Ethics Statement

The animal study was reviewed and approved by Life and Environmental Sciences Animal Care Committee, University of Calgary and adhered to the policies of the Canadian Council of Animal Care for the ethical use of animals in research.

## Author Contributions

JV and MA designed the experiments. JV conducted the experiments. JV, RM, and MA conducted data analysis and wrote the manuscript. All authors contributed to the article and approved the submitted version.

## Funding

This work was funded by Discover Grants from NSERC to both MA (RGPIN-2021-02584) and RM (RGPIN-2020-06666).

## Conflict of Interest

The authors declare that the research was conducted in the absence of any commercial or financial relationships that could be construed as a potential conflict of interest.

## Publisher's Note

All claims expressed in this article are solely those of the authors and do not necessarily represent those of their affiliated organizations, or those of the publisher, the editors and the reviewers. Any product that may be evaluated in this article, or claim that may be made by its manufacturer, is not guaranteed or endorsed by the publisher.

## References

[B1] Abe H. Honma S. Honma K. (2007). Daily restricted feeding resets the circadian clock in the suprachiasmatic nucleus of CS mice. Am. J. Physiol. Regul. Integr. Comp. Physiol. 292, R607–R615. 10.1152/ajpregu.00331.2006 16990494

[B2] Abe H. Rusak B. (1992). Anticipatory activity and entrainment of circadian rhythms in Syrian hamsters exposed to restricted palatable diets. Am. J. Physiol. 263, R116–R124. 10.1152/ajpregu.1992.263.1.R116 1636778

[B3] Antle M. C. Mistlberger R. E. (2000). Circadian clock resetting by sleep deprivation without exercise in the Syrian hamster. J. Neurosci. 20, 9326–9332. 10.1523/JNEUROSCI.20-24-09326.2000 11125012 PMC6772997

[B4] Antle M. C. Silver R. (2005). Orchestrating time: arrangements of the brain circadian clock. Trends Neurosci. 28, 145–151. 10.1016/j.tins.2005.01.003 15749168

[B5] Boulos Z. Rosenwasser A. M. Terman M. (1980). Feeding schedules and the circadian organization of behavior in the rat. Behav. Brain Res. 1, 39–65. 10.1016/0166-4328(80)90045-5 7284080

[B6] Boulos Z. Terman M. (1980). Food availability and daily biological rhythms. Neurosci. Biobehav. Rev. 4, 119–131. 10.1016/0149-7634(80)90010-X 6106914

[B7] Buhr E. D. Yoo S. H. Takahashi J. S. (2010). Temperature as a universal resetting cue for mammalian circadian oscillators. Science 330, 379–385. 10.1126/science.1195262 20947768 PMC3625727

[B8] Castillo M. R. Hochstetler K. J. Tavernier R. J. Jr. Greene D. M. Bult-Ito A. (2004). Entrainment of the master circadian clock by scheduled feeding. Am. J. Physiol. Regul. Integr. Comp. Physiol. 287, R551–R555. 10.1152/ajpregu.00247.2004 15155280

[B9] Crosby P. Hamnett R. Putker M. Hoyle N. P. Reed M. Karam C. J.. (2019). Insulin/IGF-1 drives PERIOD synthesis to entrain circadian rhythms with feeding time. Cell 177, 896–909.e820. 10.1016/j.cell.2019.02.017 31030999 PMC6506277

[B10] Dallmann R. Lemm G. Mrosovsky N. (2007). Toward easier methods of studying nonphotic behavioral entrainment in mice. J. Biol. Rhythms 22, 458–461. 10.1177/0748730407306042 17876067

[B11] Damiola F. Le Minh N. Preitner N. Kornmann B. Fleury-Olela F. Schibler U. (2000). Restricted feeding uncouples circadian oscillators in peripheral tissues from the central pacemaker in the suprachiasmatic nucleus. Genes Dev. 14, 2950–2961. 10.1101/gad.183500 11114885 PMC317100

[B12] Gibbs F. P. (1979). Fixed interval feeding does not entrain the circadian pacemaker in blind rats. Am. J. Physiol. 236, R249–253. 10.1152/ajpregu.1979.236.5.R249 443404

[B13] Hara R. Wan K. Wakamatsu H. Aida R. Moriya T. Akiyama M.. (2001). Restricted feeding entrains liver clock without participation of the suprachiasmatic nucleus. Genes Cells 6, 269–278. 10.1046/j.1365-2443.2001.00419.x 11260270

[B14] Hastings M. H. Maywood E. S. Brancaccio M. (2019). The mammalian circadian timing system and the suprachiasmatic nucleus as its pacemaker. Biology 8, 13. 10.3390/biology8010013 30862123 PMC6466121

[B15] Hughes A. T. L. Piggins H. D. (2012). Feedback actions of locomotor activity to the circadian clock. Prog. Brain Res. 199, 305–336. 10.1016/B978-0-444-59427-3.00018-6 22877673

[B16] Jha P. K. Bouâouda H. Kalsbeek A. Challet E. (2021). Distinct feedback actions of behavioural arousal to the master circadian clock in nocturnal and diurnal mammals. Neurosci. Biobehav. Rev. 123, 48–60. 10.1016/j.neubiorev.2020.12.011 33440199

[B17] Kennedy G. A. Coleman G. J. Armstrong S. M. (1991). Restricted feeding entrains circadian wheel-running activity rhythms of the kowari. Am. J. Physiol. 261, R819–827. 10.1152/ajpregu.1991.261.4.R819 1928428

[B18] Kennedy G. A. Coleman G. J. Armstrong S. M. (1996). Daily restricted feeding effects on the circadian activity rhythms of the stripe-faced dunnart, *Sminthopsis macroura*. J. Biol. Rhythms 11, 188–195. 10.1177/074873049601100301 8872591

[B19] Marchant E. G. Mistlberger R. E. (1995). Morphine phase-shifts circadian rhythms in mice: role of behavioural activation. Neuroreport 7, 209–212. 10.1097/00001756-199512000-00050 8742453

[B20] Marchant E. G. Mistlberger R. E. (1997). Anticipation and entrainment to feeding time in intact and SCN-ablated C57BL/6j mice. Brain Res. 765, 273–282. 10.1016/S0006-8993(97)00571-4 9313900

[B21] Mcfarlane H. G. Kusek G. K. Yang M. Phoenix J. L. Bolivar V. J. Crawley J. N. (2008). Autism-like behavioral phenotypes in BTBR T+tf/J mice. Genes Brain Behav. 7, 152–163. 10.1111/j.1601-183X.2007.00330.x 17559418

[B22] Mei Y. Teng H. Li Z. Zeng C. Li Y. Song W.. (2021). Restricted feeding resets endogenous circadian rhythm in female mice under constant darkness. Neurosci. Bull. 37, 1005–1009. 10.1007/s12264-021-00669-w 33779891 PMC8275728

[B23] Mendoza J. Graff C. Dardente H. Pevet P. Challet E. (2005). Feeding cues alter clock gene oscillations and photic responses in the suprachiasmatic nuclei of mice exposed to a light/dark cycle. J. Neurosci. 25, 1514–1522. 10.1523/JNEUROSCI.4397-04.2005 15703405 PMC6725981

[B24] Meyza K. Nikolaev T. Kondrakiewicz K. Blanchard D. C. Blanchard R. J. Knapska E. (2015). Neuronal correlates of asocial behavior in a BTBR T (+) Itpr3(tf)/J mouse model of autism. Front. Behav. Neurosci. 9, 199. 10.3389/fnbeh.2015.00199 26300749 PMC4526814

[B25] Meyza K. Z. Blanchard D. C. (2017). The BTBR mouse model of idiopathic autism - current view on mechanisms. Neurosci. Biobehav. Rev. 76, 99–110. 10.1016/j.neubiorev.2016.12.037 28167097 PMC5403558

[B26] Mistlberger R. E. (1991). Scheduled daily exercise or feeding alters the phase of photic entrainment in Syrian hamsters. Physiol. Behav. 50, 1257–1260. 10.1016/0031-9384(91)90592-C 1798784

[B27] Mistlberger R. E. (1994). Circadian food-anticipatory activity: formal models and physiological mechanisms. Neurosci. Biobehav. Rev. 18, 171–195. 10.1016/0149-7634(94)90023-X 8058212

[B28] Mistlberger R. E. (2005). Circadian regulation of sleep in mammals: role of the suprachiasmatic nucleus. Brain Res. Brain Res. Rev. 49, 429–454. 10.1016/j.brainresrev.2005.01.005 16269313

[B29] Mistlberger R. E. Antle M. C. (2011). Entrainment of circadian clocks in mammals by arousal and food. Essays Biochem. 49, 119–136. 10.1042/bse0490119 21819388

[B30] Mistlberger R. E. Kent B. A. Landry G. J. (2009). Phenotyping food entrainment: motion sensors and telemetry are equivalent. J. Biol. Rhythms 24, 95–98. 10.1177/0748730408329573 19150932

[B31] Mistlberger R. E. Marchant E. G. Kippin T. E. (2001). Food-entrained circadian rhythms in rats are insensitive to deuterium oxide. Brain Res. 919, 283–291. 10.1016/S0006-8993(01)03042-6 11701140

[B32] Mistlberger R. E. Sinclair S. V. Marchant E. G. Neil L. (1997). Phase shifts to refeeding in the Syrian hamster mediated by running activity. Physiol. Behav. 61, 273–278. 10.1016/S0031-9384(96)00408-8 9035258

[B33] Mrosovsky N. (1996). Locomotor activity and non-photic influences on circadian clocks. Biol. Rev. Camb. Philos. Soc. 71, 343–372. 10.1111/j.1469-185X.1996.tb01278.x 8761159

[B34] Pavlovski I. Evans J. A. Mistlberger R. E. (2018). Feeding time entrains the olfactory bulb circadian clock in anosmic PER2::LUC mice. Neuroscience 393, 175–184. 10.1016/j.neuroscience.2018.10.009 30321586

[B35] Petersen C. C. Patton D. F. Parfyonov M. Mistlberger R. E. (2014). Circadian food anticipatory activity: entrainment limits and scalar properties re-examined. Behav. Neurosci. 128, 689–702. 10.1037/bne0000017 25285457

[B36] Power S. C. Mistlberger R. E. (2020). Food anticipatory circadian rhythms in mice entrained to long or short day photoperiods. Physiol. Behav. 222, 112939. 10.1016/j.physbeh.2020.112939 32407832

[B37] Ramkisoensing A. Gu C. Van Engeldorp Gastelaars H. M. Michel S. Deboer T. Rohling J. H.. (2014). Enhanced phase resetting in the synchronized suprachiasmatic nucleus network. J. Biol. Rhythms 29, 4–15. 10.1177/0748730413516750 24492878

[B38] Rosenwasser A. M. Pelchat R. J. Adler N. T. (1984). Memory for feeding time: possible dependence on coupled circadian oscillators. Physiol. Behav. 32, 25–30. 10.1016/0031-9384(84)90064-7 6718530

[B39] Stephan F. K. (1986a). Coupling between feeding- and light-entrainable circadian pacemakers in the rat. Physiol. Behav. 38, 537–544. 10.1016/0031-9384(86)90422-1 3823166

[B40] Stephan F. K. (1986b). Interaction between light- and feeding-entrainable circadian rhythms in the rat. Physiol. Behav. 38, 127–133. 10.1016/0031-9384(86)90142-3 3786492

[B41] Stephan F. K. (1986c). The role of period and phase in interactions between feeding- and light-entrainable circadian rhythms. Physiol. Behav. 36, 151–158. 10.1016/0031-9384(86)90089-2 3952175

[B42] Stephan F. K. (2002). The “other” circadian system: food as a Zeitgeber. J. Biol. Rhythms 17, 284–292. 10.1177/074873002129002591 12164245

[B43] Stephan F. K. Swann J. M. Sisk C. L. (1979a). Anticipation of 24-hr feeding schedules in rats with lesions of the suprachiasmatic nucleus. Behav. Neural Biol. 25, 346–363. 10.1016/S0163-1047(79)90415-1 464979

[B44] Stephan F. K. Swann J. M. Sisk C. L. (1979b). Entrainment of circadian rhythms by feeding schedules in rats with suprachiasmatic lesions. Behav. Neural Biol. 25, 545–554. 10.1016/S0163-1047(79)90332-7 464989

[B45] Stokkan K. A. Yamazaki S. Tei H. Sakaki Y. Menaker M. (2001). Entrainment of the circadian clock in the liver by feeding. Science 291, 490–493. 10.1126/science.291.5503.490 11161204

[B46] Vijaya Shankara J. (2019). Characterizing circadian behaviour in the BTBR mouse model (Ph.D.), University of Calgary. 10.11575/PRISM/36817

[B47] Vijaya Shankara J. Horsley K. Cheng N. Rho J.M. Antle M.C. (in press). Circadian responses to light in the BTBR mouse. J Biol Rhythms. 10.1177/07487304221102279 PMC9452857 35722987

[B48] Wakamatsu H. Yoshinobu Y. Aida R. Moriya T. Akiyama M. Shibata S. (2001). Restricted-feeding-induced anticipatory activity rhythm is associated with a phase-shift of the expression of mPer1 and mPer2 mRNA in the cerebral cortex and hippocampus but not in the suprachiasmatic nucleus of mice. Eur. J. Neurosci. 13, 1190–1196. 10.1046/j.0953-816x.2001.01483.x 11285016

[B49] Webb I. C. Antle M. C. Mistlberger R. E. (2014). Regulation of circadian rhythms in mammals by behavioral arousal. Behav. Neurosci. 128, 304–325. 10.1037/a0035885 24773430

[B50] Yamakawa G. R. Basu P. Cortese F. Macdonnell J. Whalley D. Smith V. M.. (2016). The cholinergic forebrain arousal system acts directly on the circadian pacemaker. Proc. Natl. Acad. Sci. U. S. A. 113, 13498–13503. 10.1073/pnas.1610342113 27821764 PMC5127341

